# Diversity, palaeoecology and palaeoenvironmental significance of the Eocene chondrichthyan assemblages of the Bolca Lagerstätte, Italy

**DOI:** 10.1111/let.12436

**Published:** 2021-07-22

**Authors:** Giuseppe Marramà, Giorgio Carnevale, Jürgen Kriwet

**Affiliations:** ^1^ Dipartimento di Scienze della Terra Università degli Studi di Torino via Valperga Caluso 35 Turin 10125 Italy; ^2^ Department of Palaeontology University of Vienna Althanstrasse 14 Vienna 1090 Austria

**Keywords:** Monte Postale, Neoselachii, palaeobathymetry, Pesciara, Tethys, Ypresian

## Abstract

Over the last few years, the morphology, taxonomy and systematics of the cartilaginous fish taxa of the two main sites of the Bolca Lagerstätte, Italy, (Pesciara and Monte Postale sites) have been extensively discussed in a series of papers, resulting in a complete revision of this neglected component of the Eocene Tethyan ichthyofauna. Here, we provide a comprehensive overview of the diversity, palaeoecology and palaeoenvironmental significance of the two chondrichthyan assemblages of the Pesciara and Monte Postale sites. The assemblages include 14 shark species (Lamniformes and Carcharhiniformes) and batoids (Torpediniformes, Rhinopristiformes, Myliobatiformes, Platyrhinidae and Zanobatidae), as well as a single putative chimaeriform. The Pesciara and Monte Postale sites are characterized by eight chondrichthyan taxa each, but the taxonomic compositions are distinctly different reflecting the dissimilarities in the overall composition of both fish assemblages. Palaeoecological interpretations and habitat preferences of the two chondrichthyan assemblages are consistent with previously hypothesized palaeoenvironmental settings based on sedimentological, palaeontological and geochemical evidence. The chondrichthyan assemblages of the two sites appear to be constituted by ecologically vicariant taxa, with both characterized by a predominance of benthic species with durophagous/cancritrophic feeding modes. Taxonomic composition, habitat preferences and palaeobathymetric analyses support the hypothesis that both assemblages occupied tropical marine shallow waters (likely up to 50 m deep) of the inner portion of the Lessini Shelf. The taxonomic composition of both sites is considerably different from that of any other contemporaneous Tethyan and Boreal chondrichthyan assemblages.

The celebrated Ypresian palaeontological sites of Bolca, in northeastern Italy, are well known for the outstanding abundance, diversity and exquisite preservation of fossils, especially fishes, which provide a snapshot into the shallow marine life of the western Tethys Ocean during the early Eocene (Carnevale *et al*. 2014; Marramà *et al*. 2016a, 2016b; Friedman & Carnevale 2018). In the last four centuries of excavations, the two main sites of the Bolca Lagerstätte, Pesciara and Monte Postale yielded collectively over 500 species of fishes, terrestrial vertebrates, insects, marine invertebrates and plants. Recent studies extensively contributed to the knowledge of the geology, palaeoenvironment and taphonomy of these fossiliferous deposits (Papazzoni & Trevisani 2006; Marramà *et al*. 2016c; Papazzoni *et al*. 2017), as well as to the taxonomic diversity of the fishes, so far including more than 250 species‐level taxa (see Carnevale *et al*. 2014; Friedman & Carnevale 2018). However, despite the considerable efforts devoted to the study of fossil fishes, the diversity, palaeoecology and palaeoenvironmental significance of certain groups, particularly the cartilaginous fishes (sharks, rays and chimaeras), have been overlooked until recently. The last comprehensive account focussing on these fishes was provided at the end of 19th century by Jaekel (1894). Except for revisions of selected taxa by Cappetta (1975), Carvalho (2010) and Fanti *et al*. (2016), a number of detailed re‐evaluations of the taxonomy, systematics and palaeobiology of the Bolca Lagerstätten chondrichthyans have been carried out by the authors of this paper in the last years (Marramà *et al*. 2018a–c, 2019a–d, 2020a, 2020b, 2020c).

The goal of this paper is to conclusively assess and summarize the diversity, palaeoecological role and palaeoenvironmental significance of the chondrichthyan assemblages of the Pesciara and Monte Postale sites, based on comparisons with the feeding and habitat preferences of extant closely related species.

## Geological setting

The fossiliferous sites of the Pesciara and Monte Postale are the most productive deposits of the Bolca Lagerstätten, which lie on the eastern part of Monti Lessini, in northern Italy. Although the two sites are about 300 m from each other and share similar sedimentological features, such as finely laminated micritic limestones with fish and plant remains, they differ from a stratigraphical, palaeontological, palaeoenvironmental and taphonomic point of view (Papazzoni & Trevisani 2006; Papazzoni *et al*. 2014a, 2014b, 2017; Marramà *et al*. 2016c).

The Pesciara site has been exploited since the mid‐16th century. It consists of a 20‐m‐thick limestone block surrounded by volcanic deposits and formed by a cyclic alternation of finely laminated micritic limestone with fishes and plants and grainstone bearing benthic fossils (Papazzoni & Trevisani 2006). Based on the larger benthic foraminiferan content, the fish‐bearing limestone of the Pesciara site has been referred to the *Alveolina dainellii* Zone, corresponding to the uppermost part of the Shallow Benthic Zone (SBZ) 11 (late Ypresian; 48.96–48.50 Ma) (Papazzoni & Trevisani 2006). Conversely, the succession of Monte Postale consists of more than 130 m of grainstone alternating with coralgal limestone and laminated wackestone with fishes and plants (Vescogni *et al*. 2016; Papazzoni *et al*. 2017). The Monte Postale spans the entire NP 13 and CNE 5 calcareous nannoplankton zones corresponding to a large part of the SBZ 11 in the time interval of between 50.50 and 48.96 Ma (Papazzoni *et al*. 2017).

Controlled excavations carried out at the Pesciara and Monte Postale sites show that the taxonomic composition of their fish assemblages and taphonomic features differ between the two sites as a consequence of the different physiography and environmental conditions of the original palaeobiotopes (Marramà *et al*. 2016c). Fossils from the Pesciara site are usually complete and exquisitely preserved, suggesting a rapid accumulation and burial of the carcasses over a poorly oxygenated substrate. The development of microbial biofilms promoted the high‐quality preservation of the fossils protecting them from decomposition, scavenger activity and bottom currents (Marramà *et al*. 2016c). Moreover, several fish specimens exhibit the typical features of muscular tetany, suggesting that toxic algal blooms might represent one of the main causes of death of marine organisms from Pesciara (Marramà *et al*. 2016c). Conversely, the fossils from Monte Postale are mostly incomplete and strongly disarticulated, and several fishes show disruption of fins, S‐shaped curving of the vertebral column and unidirectional dispersion of scales around the body, thereby indicating an episodic disturbance of the bottom that promoted periodic aerobic conditions, as suggested by the presence of a diverse benthic fauna and bioturbation tracks (Marramà *et al*. 2016c).

## Material and methods

**Fig. 1:**
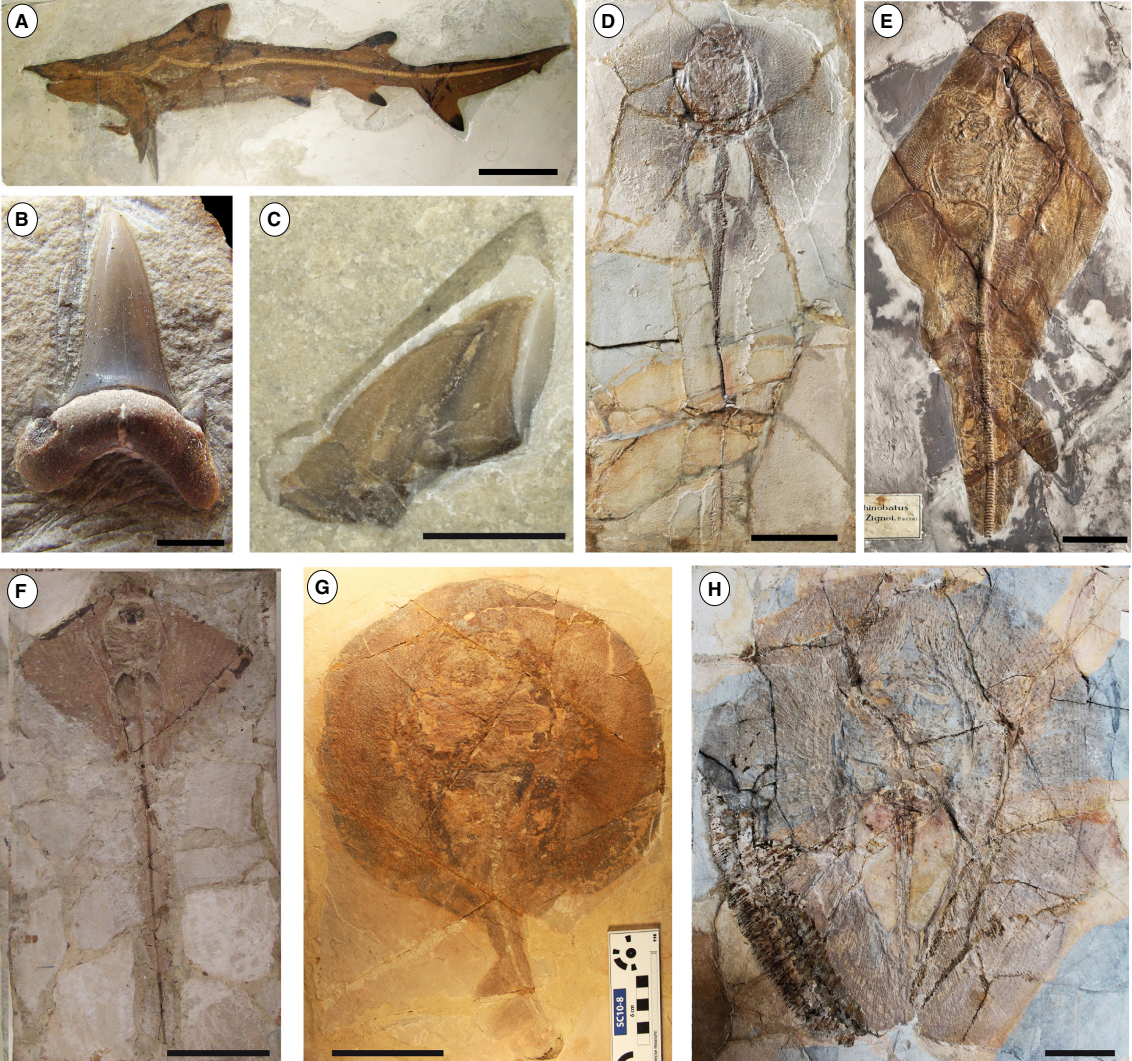
Chondrichthyan taxa from the Pesciara site: A, *Galerorhinus cuvieri*, MCSNV T.1124. B, *Brachycarcharias lerichei*, MCSNV IG.135779, isolated tooth. C, *Eogaleus bolcensis*, MCSNV T.415, isolated tooth. D, *Tethytrygon muricatus*, MNHN F. Bol564, holotype. E, *Eorhinobatos primaevus*, MGP‐PD 26278, holotype. F, *Promyliobatis gazolai*, MCSNV VII.B.90, holotype. G, *Plesiozanobatus egertoni*, MB.f 1608.1. H, *Lessiniabatis aenigmatica*, MNHN F. Bol.566, holotype. Scale bars: A, D–H = 100 mm; B–C = 2 mm.

**Fig. 2:**
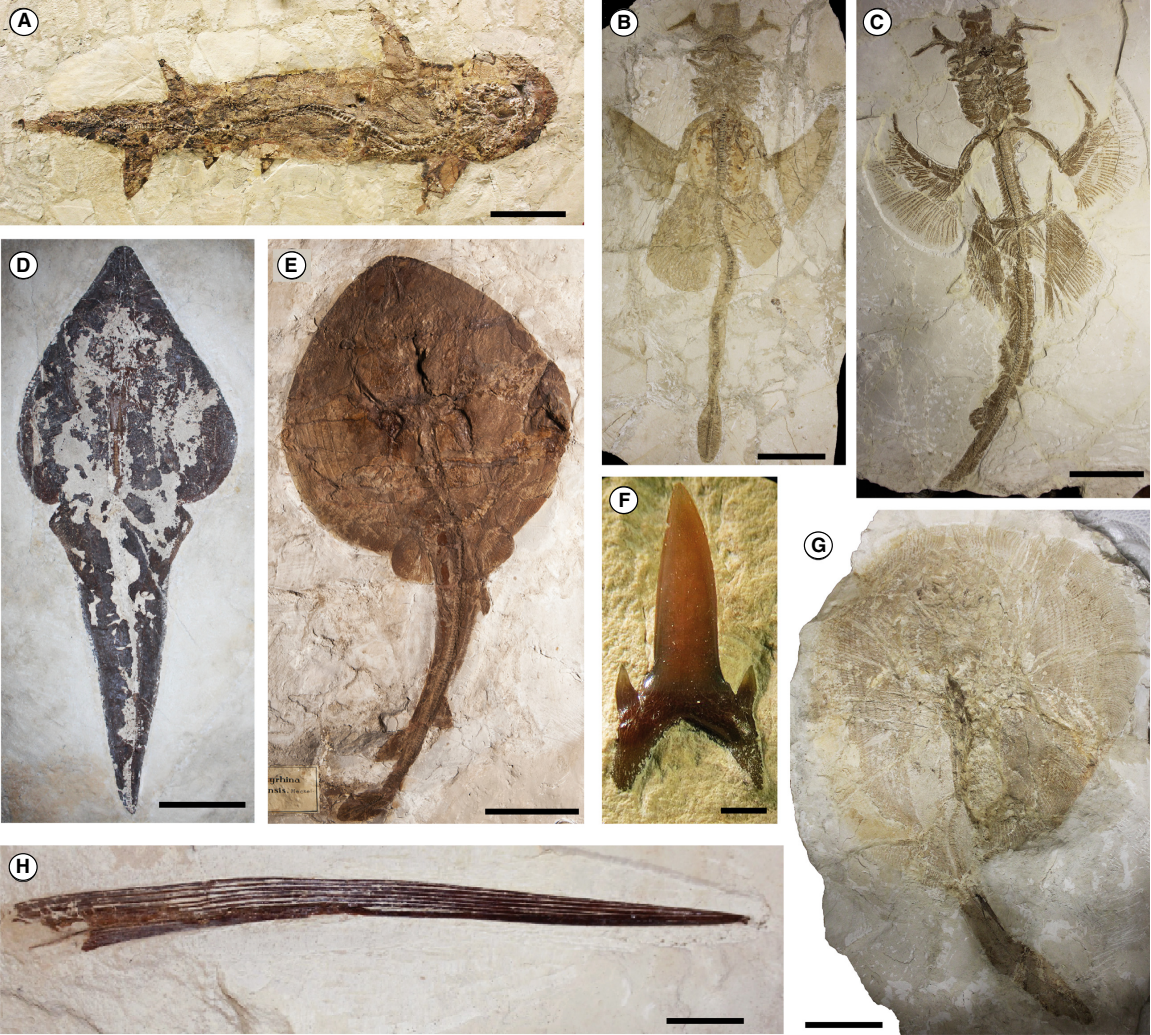
Chondrichthyan taxa from the Monte Postale site: A, *Eogaleus bolcensis*, MCSNV T.311, holotype. B, *Titanonarke megapterygia*, MCSNV IG.135576, holotype. C, *Titanonarke molini*, MCSNV IG.VR.67290. D, *Pseudorhinobatos dezigni*, NHMW 1853.XXVII.4, holotype. E, *Eoplatyrhina bolcensis*, MGP‐PD 26279C. F, *Brachycarcharias lerichei*, MCSNV IG.VR.69800, isolated tooth. G, *Arechia crassicaudata*, MCSNV IG.VR.27607, neotype. H, *Ischyodus* sp., dorsal‐fin spine, MCSNV IG.VR.61511. Scale bars: A–E, G = 100 mm; F = 2 mm; H = 20 mm.

Four centuries of excavations at the Bolca Lagerstätten yielded at least 68 chondrichthyan specimens from the Pesciara and Monte Postale sites (Table 1; Figs 1, 2). The specimens are kept in museums and institutions worldwide, including the Museo Civico di Storia Naturale di Verona (MCSNV), Museo dei Fossili di Bolca, Museo di Geologia e Palaeontologia dell'Università di Padova (MGP‐PD), Museo di Storia Naturale di Milano (MSNM), Museum National d'Histoire Naturelle, Paris (MNHN), Museo Friulano di Storia Naturale di Udine (MFSN), Museo di Storia Naturale dell'Università di Firenze (MSNFI), Museo di Storia Naturale dell'Università di Pavia (MSNPV), Museo Geologico Giovanni Capellini dell'Università di Bologna (MGGC), Museum für Naturkunde Berlin (Mf.B), Naturhistorisches Museum Wien (NHMW), Carnegie Museum, Pittsburgh (CMNH), Museum of Comparative Zoology, Harvard University (MCZ) and Natural History Museum, London (NHMUK).

**Table 1:** Habitat and feeding preferences of the Pesciara and Monte Postale chondrichthyan taxa, based on the biology of their extant relatives.

Site	Superorder	Order	Family	Taxon	N.	Habitat		Dentition type	Diet				
						Benthic	Benthopelagic		Piscivorous	Durophagous/ cancritrophic	Teuthitrophic	Polychaetes	Foraminifera
Pesciara	Galeomorphii	Carcharhiniformes	Triakidae	*Galeorhinus cuvieri*	6			Cutting–clutching	X*	X	X		
			Carcharhinidae	*Eogaleus bolcensis*	1		?	Cutting–clutching	X	X			
		Lamniformes	Odontaspididae	*Brachycarcharias lerichei*	7			Tearing	X		?		
	Batomorphii	Myliobatiformes	Dasyatidae	*Tethytrygon muricatus*	13			Crushing–clutching	X*	X		X	
			‘Myliobatidae’	*Promyliobatis gazolai*	2			Grinding	X	X		X	
			Incertae Sedis	*Lessiniabatis aenigmatica*	3			Crushing–clutching	?	X			
		Rhinopristiformes	Rhinobatidae	*Eorhinobatos primaevus*	1			Crushing	X	X			
		Incertae sedis	Zanobatidae	*Plesiozanobatus egertoni*	6			Crushing–clutching	X	X			
Monte Postale	Galeomorphii	Carcharhiniformes	Carcharhinidae	*Eogaleus bolcensis*	4		?	Cutting–clutching	X	X			
		Lamniformes	Odontaspididae	*Brachycarcharias lerichei*	8			Tearing	X		?		
	Batomorphii	Torpediniformes	Narcinidae	*Titanonarke molini*	5			Clutching	X	X		X	X*
				*Titanonarke megapterygia*	1			Clutching	X	X		X	
		Myliobatiformes	Urolophidae	*Arechia crassicaudata*	6			Crushing–clutching	X*	X*		X	
		Rhinopristiformes	Rhinobatidae	*Pseudorhinobatos dezignii*	1			Crushing	X	X			
		Incertae sedis	Platyrhinidae	*Eoplatyrhina bolcensis*	3			Crushing	X	X			
	Holocephalimorpha	Chimaeriformes	Callorhynchidae	*Ischyodus* sp.	1			Crushing		X			

Grey cells indicate the habitat and feeding preferences. Asterisks indicate that diet is inferred from direct observation (gut content). Dentition type terminology is based on Cappetta (2012). Habitat and dietary preferences of extant families used to infer diet of the taxa recorded in the Pesciara and Monte Postale sites are mainly compiled from Cortés *et al*. (2008), Last *et al*. (2016) and the FishBase website (Froese & Pauly 2019).

In this paper, the term ‘diversity’ stands for species richness, that is, number of species. Where the identification was established only at the genus level (e.g. *Ischyodus*), we consider that at least one species was present. Although all the chondrichthyan species of the two Bolca assemblages are extinct, we inferred their dietary preferences directly by analysing their dental type (see Cappetta 2012), by direct observations of their fossilized gut content and by comparison with the ecology of the closest representatives of the extant families they belong to. Although extant chondrichthyans have wide ranges of diet, each family has specific food preferences (Cortés 1999; Cortés *et al*. 2008) that could be used to infer dietary preferences of their fossil relatives (e.g. Carrillo‐Briceño *et al*. 2016, 2019). Dietary preferences of extant families used to infer diet of the fossil taxa (Table 1) were mainly compiled from Cortés *et al*. (2008), Last *et al*. (2016) and the FishBase website (Froese & Pauly 2019). Habitat and bathymetric preferences of extant chondrichthyan families were used to infer those of the fossil taxa, mainly following Last *et al*. (2016) and the FishBase website (Froese & Pauly 2019).

We also compared the taxonomic composition of the chondrichthyan assemblages of the Pesciara and Monte Postale sites with those of other Eocene Boreal and Tethyan deposits by using a comprehensive literature data set (Marramà *et al*. 2021). Boreal chondrichthyan assemblages include the following: the Ypresian sediments of London Clay Formation in England, U.K. (47 genera; Cooper 1977; Rayner *et al*. 2009) and Paris basin in France (45 genera; Dutheil *et al*. 2006; Adnet & Cappetta 2008); the middle Lutetian Lede Sand Formation in Belgium (54 genera; Nolf 1988; Eeckhaut & De Schutter 2009) and Fürstenau Formation in northern Germany (18 genera; Diedrich 2012); the Ypresian‐Lutetian of Lillebælt Clay in Denmark (31 genera; Carlsen & Cuny 2014). Tethyan chondrichthyan assemblages include those from the Ypresian‐Lutetian southwestern France (32 genera; Adnet 2006; Adnet *et al*. 2008) and Northern Morocco (29 genera; Noubhani & Cappetta 1997); the Bartonian‐Priabonian Ad‐Dakhla region of southwestern Morocco (38 genera; Adnet *et al*. 2010) and Midawara Formation of the Fayum area in Egypt (34 genera; Underwood *et al*. 2011); and two Ypresian localities from India, the Cambay Shale (11 genera; Rana *et al*. 2004) and Kapurdi Formation (nine genera; Rana *et al*. 2006).

Faunal comparisons were assessed using the Sørensen–Dice similarity index (Dice 1945; Sørensen 1948), which measures the similarity between two biological communities based on presence/absence data. We used this index because it is one of the most commonly used indices for living and fossil communities and is regarded as one of the most effective measures of similarity based on presence/absence (Southwood & Henderson 2000; Magurran 2004; Villafaña *et al*. 2020). Analyses were carried out at the genus level. Although the Sørensen–Dice index is sensitive to the completeness of taxonomic inventories (Jost *et al*. 2011), we assume that the taxonomic diversity of the cartilaginous fish assemblages of the Bolca Lagerstätten is complete, because the abundance and high‐quality preservation of the fossil fishes recovered in these conservation deposits likely reflect a real biological and ecological signal (Marramà *et al*. 2018a). We also tested the faunal similarities by using cluster analysis and non‐metric multidimensional scaling in order to recognize associations hierarchically grouped together reflecting their palaeobiogeographical and palaeoenvironmental similarities.

## Results

### Taxonomic composition

The overall taxonomic composition of the two fossiliferous localities includes 14 species belonging to 13 genera pertaining to 11 families and six orders of galeomorphs, batomorphs and holocephalans (Table 1). All of these orders and families are still in existence today, but all the genera are extinct, except for *Galeorhinus*. The most remarkable feature is the very limited taxonomic overlap between the Pesciara and Monte Postale assemblages, with the sharks *Brachycarcharias lerichei* and *Eogaleus bolcensis* being the only taxa shared by both sites (Table 1; Figs 1, 2). Squalomorph sharks and skates (the Rajiformes sensu Naylor *et al*. 2012) have not been recorded in the Bolca palaeobiotopes.

#### Pesciara site

Galeomorph sharks of the Pesciara site are represented by three genera in three families of the orders Carcharhiniformes and Lamniformes. Carcharhiniforms include *Galeorhinus cuvieri* (family Triakidae) that is represented by six specimens from the Pesciara site, which were interpreted to document a variety of ontogenetic stages of juvenile individuals (Fanti *et al*. 2016). Teeth of this shark species were proposed to belong to the extinct carcharhinid *Physogaleus* by Adnet & Cappetta (2008) but other morphological traits (e.g. denticle morphology) typical of triakid sharks seem to contradict this hypothesis. Another shark, *E. bolcensis* (Carcharhinidae), is represented by a single tooth (Marramà *et al*. 2018b). Isolated teeth of *Eogaleus* have been reported from the Eocene of China (Li 1997) and India (Rana *et al*. 2004), although these occurrences are doubtful (Marramà *et al*. 2018b). However, the palaeobiogeographical distribution of *Eogaleus* might be wider and not only limited to the Bolca area. In fact, it has been hypothesized that the widespread extinct carcharhinid *Physogaleus* might be considered a junior synonym of *Eogaleus*, based on the similar tooth morphology and the fact that the skeletal remains of *Eogaleus* occur within the Ypresian palaeobiogeographical distribution area of *Physogaleus* (Marramà *et al*. 2018b). Lamniform sharks are the only chondrichthyans from Bolca uniquely represented by isolated teeth. Their combination of characters supported their assignment to the extinct odontaspidid *B. lerichei*, a species widely spread across the North Hemisphere during the early Palaeogene (Marramà *et al*. 2019a). Seven teeth of *B. lerichei* have been collected from the Pesciara site to date.

Stingrays of the order Myliobatiformes are the most diverse batoid clade. At the Pesciara site, whiptail stingrays of the family Dasyatidae are represented by 13 specimens of *Tethytrygon muricatus*. This taxon was described originally by Volta (1796) and recently revised by Marramà *et al*. (2019b). ‘*Dasyatis*’ *zigni*, traditionally considered the second dasyatid species, is regarded as junior synonym of *T. muricatus* (Marramà *et al*. 2019b). Morphological characters concur to suggest that *Tethytrygon* is closely related to modern *Taeniura* and *Neotrygon* of the subfamily Neotrygoninae (Marramà *et al*. 2019b). *Promyliobatis gazolai* from the Pesciara site is the only durophagous benthopelagic stingray from Bolca and represents a stem taxon to modern Myliobatidae, Aetobatidae, Rhinopteridae and Mobulidae (Marramà *et al*. 2019c). The morphological analysis of three previously undescribed specimens from the Pesciara deposit revealed the existence of a peculiar stingray, *Lessiniabatis aenigmatica*, which is unique among the myliobatiforms in its body plan making its referral to a known stingray family difficult (Marramà *et al*. 2019d). Rhinopristiforms are represented by a single specimen of an extinct rhinobatid, *Eorhinobatos primaevus* (Marramà *et al*. 2020c). Finally, *Plesiozanobatus egertoni* is the first known panray (family Zanobatidae) in the fossil record (Marramà *et al*. 2020b).

#### Monte Postale site

Carcharhiniforms include *E. bolcensis* (Carcharhinidae), represented by four articulated adult or subadult individuals (Marramà *et al*. 2018b; Larocca Conte *et al*. 2020), whereas lamniforms are represented by eight isolated teeth of *B. lerichei*. At Monte Postale, stingrays are represented by six specimens of *Arechia crassicaudata*, a large‐sized stingaree species (Urolophidae) (Marramà *et al*. 2020a). Electric rays of the order Torpediniformes are represented by five individuals of *Titanonarke molini* and a single specimen of *Titanonarke megapterygia*. The revision of the Eocene electric rays provided by Marramà *et al*. (2018c) revealed outstanding information about their palaeobiology, particularly about diet preferences, whereas the study of Robin *et al*. (2019) shed light on their biological interactions with isopod crustaceans. Rhinopristiform guitarfishes are represented by a single specimen of the rhinobatid *Pseudorhinobatos dezignii* (Marramà *et al*. 2020c). The recent revision by Marramà *et al*. (2020b) revealed that the thornback ray family Platyrhinidae is only represented by *Eoplatyrhina bolcensis*. Finally, a single dorsal‐fin spine from Monte Postale has been tentatively referred to the extinct callorhynchid chimaeriform *Ischyodus* (see Marramà *et al*. 2018a), whose fossil record spreads from middle Jurassic to Pliocene according to Stahl (1999).

### Palaeobathymetry

**Fig. 3:**
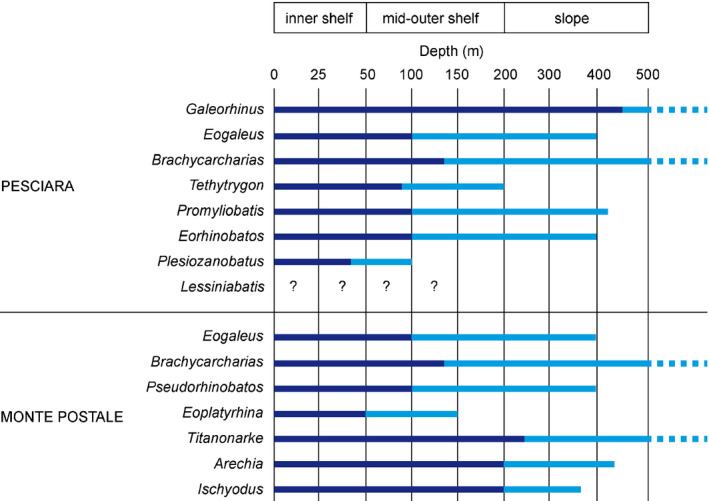
Bathymetric ranges estimated for the taxa of the Pesciara and Monte Postale palaeobiotopes based on the biology of their extant closest relatives. Dark blue indicates the most probably preferred range. Dashed lines indicate that the bathymetric range is greater. Question marks indicate that depth range for *Lessiniabatis* cannot be inferred, being difficult to accommodate it in any known stingray family.

Both the Pesciara and Monte Postale chondrichthyan assemblages are characterized by a predominance of benthic over the benthopelagic taxa (Table 1). Based on bathymetric distributions of closer extant relatives, the most probable depth for the Pesciara palaeobiotope was between 0 and 40 m (Fig. 3). This bathymetric interval is primarily constrained by the presence of the zanobatid *Plesiozanobatus*, whose closer living relatives, *Zanobatus schoenleinii* and *Z. maculatus*, inhabit shallow waters preferably up to 40 m, although some species were rarely reported to occur at depths up to 100 m (Last *et al*. 2016; Séret 2016). All the other batoid taxa support this scenario because their extant representatives prefer waters up to 100 m within coastal environments. In the Pesciara assemblage, the most abundant chondrichthyan taxon is the neotrygonine *T. muricatus*. Extant stingrays of the subfamily Neotrygoninae are demersal, benthic marine batoids occurring inshore on continental or insular shelves at depths up to 90 m, although some species also were found offshore to 200 m and inhabiting warm–temperate and tropical shallow waters often associated with coral reefs (Last *et al*. 2016). Apparently, the presence of the triakid *Galeorhinus* and the odontaspidid *Brachycarcharias* seem to contrast with this palaeobathymetric scenario because extant relatives have a wider depth distribution and are able to move significant distances over oceanic basins. For instance, the extant *Galeorhinus galeus* and the odontaspidid genus *Odontaspis* occur from the surface to the outer shelves and down the slopes to possibly 1600 m, whereas *Carcharias* (the only other living representative of the para‐ or polyphyletic family Odontaspididae) is observed from 0 to 131 m depth, but mostly occurs between 15 and 25 m (Compagno 1984a, 1984b, 2003; Cappetta *et al*. 2019; Froese & Pauly 2019). However, extrapolating the palaeoecology of *G. cuvieri* and *B. lerichei* from only three extant sharks might represent a limitation because some triakids and odontaspidids in the past may have had different habitat preferences (e.g. *Sylvestrilamia* seems to be limited to brackish waters). Moreover, it is important to take into account that all the *Galeorhinus* individuals from the Pesciara site are interpreted to be juveniles (Fanti *et al*. 2016). It was suggested that the presence of juvenile individuals of *Galeorhinus* in the Pesciara palaeobiotope might be related, at least in part, to the competitive advantage of juvenile shark in having access to relatively competitor‐free trophic niches and food resources in the shallow water palaeobiotopes that were probably unavailable for adult individuals (Marramà *et al*. 2019a) and probably indicate a nearby nursery area of this shark (Fanti *et al*. 2016). At the same time, the fact that *Brachycarcharias* is represented only by isolated teeth might suggest that this taxon was merely an adventitious visitor of the tropical reef‐associated palaeobiotopes of Bolca (Marramà *et al*. 2019a).

The most probable depth for the Monte Postale palaeobiotope is consistent with that of the Pesciara, ranging between 0 and 50 m, which is mainly inferred by the presence of the thornback ray platyrhinid *Eoplatyrhina*, whose living relatives, *Platyrhina* and *Platyrhinoidis*, prefer shallow waters up to 50 m, although individuals have been reported up to 150 m depth (Compagno & Last 1999a; Iwatsuki *et al*. 2011; Last *et al*. 2016). All the other chondrichthyan taxa from Monte Postale support this scenario because their extant relatives mostly inhabit shallow waters and none of them is indicative of deep waters.

In conclusion, based on the bathymetric distribution of extant relatives, our analysis suggests that both the Pesciara and Monte Postale palaeobiotopes were likely characterized by depths reaching 40–50 m, thereby supporting the assumption of a shallow‐water inner shelf scenario previously hypothesized for both sites by a number of studies (e.g. Landini & Sorbini 1996; Papazzoni & Trevisani 2006; Schwark *et al*. 2009; Papazzoni *et al*. 2014a, 2014b, 2017).

### Dietary preferences

Two main feeding preferences can be recognized in the Bolca palaeobiotopes (Table 1). In both sites, durophagous/cancritrophic taxa (mollusc and crustacean feeders) represent the most diverse group, which is dominated by batoids (mainly stingrays) and corresponds to about 63% and 50% of the taxa in the Pesciara and Monte Postale assemblages, respectively. The neotrygonine *T. muricatus* is by far the most abundant taxon of this group in the Pesciara association. The diet of extant neotrygonines mainly relies on crustaceans and bivalves, and rarely on small bony fishes and worms (Last *et al*. 2016). In the Monte Postale site, invertebrativores are mainly represented by urolophids (*Arechia*) and platyrhinids (*Eoplatyrhina*) whose living representatives feed mainly on crustaceans and augment their diet with small benthic fishes and polychaete worms (Last & Compagno 1999a; Last *et al*. 2016). Direct evidences of crustaceans as prey item include a single chela of a small decapod as gut content in one of the *Arechia* specimens (Marramà *et al*. 2020a).

The second most diverse group in both the sites is represented by piscivores, particularly juvenile triakids, small‐sized carcharhinids and odontaspidids. Piscivorous taxa represent about 37% of the chondrichthyan species in the Pesciara and 28% of the species of the Monte Postale assemblage, respectively. Evidences of piscivory include the presence of bony fishes as stomach content in *G. cuvieri* (Fanti *et al*. 2016) and some stingrays (*Arechia* and *Tethytrygon*; Marramà *et al*. 2019b, 2020a) suggesting that bony fishes were a significant component of the diet of both sharks and rays.

A third group, the benthic soft prey feeders, is only found at the Monte Postale site and is represented by two species of the extinct narcinid *Titanonarke*. Modern numbfishes prey upon benthic soft invertebrates, mostly polychaete worms, using their highly specialized protrusible feeding apparatus, although crustaceans, molluscs and small bony fishes can also be part of their diet (Carvalho *et al*. 1999; Last *et al*. 2016; Froese & Pauly 2019). Interestingly, a stomach content formed by a massive accumulation of hundreds of larger benthic foraminifera of the genus *Alveolina* was found in an individual of *T. molini*, indicating that this numbfish preyed upon foraminifera at least occasionally (Marramà *et al*. 2018c). To the best of our knowledge, there is no evidence that this feeding behaviour has evolved in other extinct or living chondrichthyans.

There are no large opportunistic eurytrophic predators (diet mostly based on fishes and other vertebrates) or microphagous filter feeders (diet based mainly on plankton) in the Bolca assemblages. The absence of such taxa can be related, at least in part, to the absence of specific food items and/or to the overall palaeoenvironmental conditions of the shallow‐water palaeobiotopes of Bolca that precluded the access to these groups, today represented by large‐sized pelagic sharks (e.g. *Galeocerdo*, *Rhincodon*) and rays (e.g. *Mobula*).

Exclusively teuthitrophic species (cephalopod feeders) appear absent, although *G. cuvieri* and *B. lerichei*, similarly to modern relatives (e.g. Compagno 1984a), might have expanded their diet to squids, which are also found at Bolca (Giusberti *et al*. 2014). However, in absence of direct evidence, this is speculative.

### Faunal comparisons

The comparative analysis of the taxonomic composition reveals little taxonomic overlap between the Bolca sites and other nearly coeval Tethyan and Boreal chondrichthyan assemblages. Although the Pesciara and Monte Postale sites share similar sedimentological features and a similar diversity (eight taxa each), their taxonomic composition is remarkably different (Dice correlation 0.267; Marramà *et al*. 2021), with *B. lerichei* and *E. bolcensis* being the only taxa shared by the two sites. The remarkably different taxonomic composition of these two chondrichthyan assemblages reflects the dissimilarities in the overall fish composition of the Pesciara and Monte Postale assemblages (see Marramà *et al*. 2016c).

The taxonomic composition of the Pesciara and Monte Postale sites is even more different from that of any other Tethyan and Boreal locality. There is very small similarity between the Pesciara fauna and those of the Kapurdi Formation (0.118) and Cambay Shale (0.105) in India, the Midwara Fm (0.095) in Egypt and SW Morocco (0.087), and between the Monte Postale fauna and that of North Morocco (0.111), North Germany and the Paris Basin (both 0.080).

**Fig. 4:**
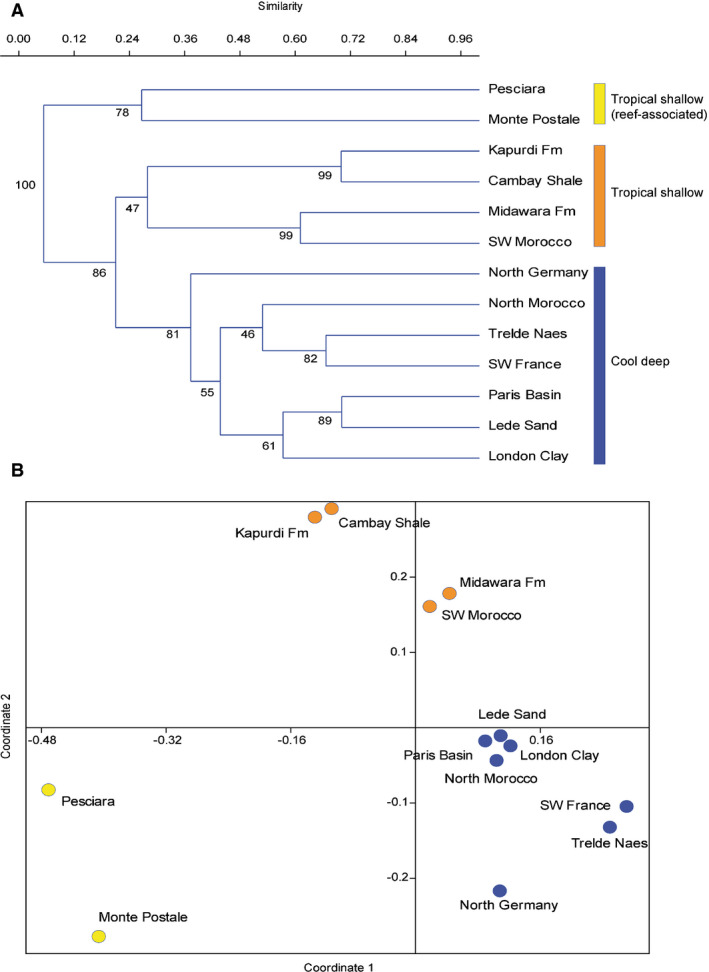
A, cluster analysis showing all chondrichthyan assemblages hierarchically grouped together as to reflect their palaeobiogeographical and palaeoenvironmental similarity; numbers on each node are bootstrap values (1000 replicates). B, non‐metric multidimensional scaling plot showing the same pattern. The Sørensen–Dice index was selected in both analyses because we use presence/absence data.

The cluster analysis shows two, fairly well‐separated clusters (Fig. 4A), one of which constitutes the Pesciara and the Monte Postale assemblages, and a second one comprising all the other assemblages, thereby evidencing the remarkable differences between Bolca and the other assemblages. In turn, two clusters can be recognized in the group including all the other Boreal and Tethyan assemblages. The first one comprises the Boreal assemblages of the London Clay, Paris Basin, North Germany, Denmark and Belgium, which are dominated by deep‐water or cool shallow genera like *Centrophorus*, *Chlamydoselachus*, *Coupatezia*, *Echinorhinus*, *Heptranchias*, *Hexanchus*, *Pristiophorus* and *Striatolamia* (Nolf 1988; Dutheil *et al*. 2006; Adnet & Cappetta 2008; Eeckhaut & De Schutter 2009; Rayner *et al*. 2009; Diedrich 2012; Carlsen & Cuny 2014). Although from a palaeobiogeographical point of view, the SW France and North Morocco deposits originated in the Tethyan realm, with the former representing a very deep‐water basin and the latter a moderately deep high nutrient shelf, the dominance of deep‐water genera such as *Chlamydoselachus*, *Heptranchias*, *Hexanchus*, *Centrophorus*, *Echinorhinus*, *Coupatezia* and *Pristiophorus* suggests deposition in cool, deep waters (Noubhani & Cappetta 1997; Adnet 2006; Adnet *et al*. 2008; Carlsen & Cuny 2014). Consequently, they are grouped in the same cluster as the Boreal assemblages (Fig. 4A).

Conversely, the other cluster includes most of the other Tethyan localities (SW Morocco, Egypt, and India), which are regarded as tropical shallow marine environments in proximity to emerged coastal areas (Rana *et al*. 2004, 2006; Adnet *et al*. 2010; Underwood *et al*. 2011). Their similarity is due to the presence of several demersal taxa, some of which mostly occur in shallow waters, especially the predatory sharks such as the carcharinids *Galeocerdo* and *Carcharhinus*, the triakid *Galeorhinus* and the odontaspidid *Brachycarcharias*, along with some pristid and myliobatiform batoids (Rana *et al*. 2004, 2006; Adnet *et al*. 2010; Underwood *et al*. 2011). The non‐metric multidimensional scaling plot supports the same pattern (Fig. 4B).

## Discussion

### Diversity significance

The chondrichthyan assemblages of the Pesciara and Monte Postale sites solely comprise extinct species (Table 1). All the genera are also extinct, except for *Galeorhinus*, which today is represented by *G. galeus*. Although the Pesciara and Monte Postale assemblages show a similar number of species, they are characterized by a different taxonomic composition. *Galeorhinus*, *Tethytrygon*, *Promyliobatis*, *Lessiniabatis*, *Eorhinobatos* and *Plesiozanobatus* are only present at the Pesciara site, whereas *Titanonarke*, *Arechia*, *Pseudorhinobatos*, *Eoplatyrhina* and *Ischyodus* are only found at the Monte Postale site. *Brachycarcharias* and possibly *Eogaleus* are present in both sites. Although Pesciara and Monte Postale represent tropical coastal marine shallow‐water environments in some ways influenced by a coral reef system, their low taxonomic similarity appears to be largely related to the different palaeoenvironmental and palaeoecological conditions, which were also responsible for the substantial differences in the bony fish fauna composition and taphonomic features (Papazzoni & Trevisani 2006; Carnevale *et al*. 2014; Marramà *et al*. 2016c; Papazzoni *et al*. 2017).

The comparison between the Bolca chondrichthyan faunas and those of other (almost) coeval Boreal and Tethyan localities (Fig. 4) provide a more complete perspective about the diversity of the Bolca cartilaginous fauna and we can infer three main considerations:

1. Bolca is one of the less diverse Eocene chondrichthyan assemblages even though its ichthyofauna is regarded as one of the most diverse fossil fish assemblages of the world, with more than 250 fish species in at least 190 genera (Carnevale *et al*. 2014). However, most of this diversity is due to the extraordinary abundance of bony fishes, which include a variety of non‐acanthomorph teleosts (e.g. osteoglossomorphs, elopomorphs, clupeomorphs, ostariophysans and aulopiforms), including some of the last representatives of the crossognathiforms and pycnodontiforms. The bony fish fauna, however, is taxonomically dominated by acanthomorph fishes, with percomorphs making up the vast majority of the fish diversity with about 160 species‐level taxa (Carnevale *et al*. 2014; Friedman & Carnevale 2018). The extraordinary diversity of the bony fish fauna of the Bolca Lagerstätten is therefore in strong contrast with the low number of chondrichthyans (14 species in total; eight species per site), which represent 5–6% of the total fish diversity. Surprisingly, this low percentage strongly resembles that of chondrichthyan species in some modern tropical shallow fish assemblages (Manilo & Bogorodsky 2003; Marranzino 2013; Great Barrier Reef Marine Park Authority 2021). Linking this to the abundance and high‐quality preservation of the fossil fishes recovered in the productive strata of the Bolca Lagerstätten, we can assume that the low diversity of cartilaginous fishes is a genuine biological and ecological signal, not a result of collection or taphonomic bias. In fact, in modern‐day environments, the majority of sharks and rays species are mid‐ to high‐order predators, and some occupy ecological niches at the top of the food chain (e.g. Wetherbee & Cortes 2004). Consequently, chondrichthyans are less common than prey species down in the trophic web and have a significant effect on the balance of the ecosystems because they regulate the prey populations (Salini *et al*. 1992; Friedlander & deMartini 2002; Dulvy *et al*. 2008; Heithaus *et al*. 2010; Bornatowski *et al*. 2014). A similar pattern is revealed by the chondrichthyans of the Bolca Lagerstätten with the majority representing 3rd level predators (e.g. durophagous batoids). While carcharhiniforms and lamniforms normally represent 4th level predators, their juvenile and small adult representatives in the Bolca Lagerstätten certainly occupied lower trophic levels.
2. The total number of Bolca individuals is very low (see Table 1) compared with the other tooth‐based assemblages (hundreds to thousands of teeth), where, in the absence of associated dentitions, it is likely that one tooth equated to one individual. From this perspective, the Bolca and the other faunas are difficult to compare. Likewise, as whole skeletons are present, it is likely that all the Bolca specimens in the collection sample have been collected, so that, unlike tooth‐based faunas, there is no collection bias. As a result, the Bolca assemblages may include species with teeth that would not normally be collected because in some sites (such as the Moroccan phosphorites), samples are only normally sorted down to a 1 mm mesh due to the grains in the sediment, and teeth of less than 2mm are, therefore, rarely seen. Conversely, large teeth and denticles from tooth‐based sites are easily found in the field but are likely to have come from numerically rare species.
3. Low taxonomic overlap between Bolca and coeval Boreal and Tethyan assemblages. The revision of the Bolca species traditionally referred to *Dasyatis*, *Urolophus*, *Rhinobatos* and *Platyrhina* revealed the existence of considerable differences between extinct and extant species, resulting in separate generic placements (Marramà *et al*. 2019b, 2020a, 2020b, 2020c). The cosmopolitan *B. lerichei* is the only species shared with other assemblages, whereas at genus level, *Ischyodus*, *Galeorhinus* and *Arechia*, are present in a few other assemblages. Therefore, the majority of the chondrichthyan taxa of the Bolca assemblages appear exclusive. It is difficult to assess whether this represents a real biological signal or is due to taphonomic and collecting biases. The Bolca sites are some of the few Palaeogene deposits where cartilaginous fishes are represented by exquisitely preserved and articulated skeletons, whereas in the other Eocene concentration, Lagerstätten chondrichthyans are represented by teeth only, which are less informative from a systematic point of view. The exquisite preservation of the Bolca specimens may have favoured the identification of diagnostic skeletal characters useful for the creation of new genera. The taphonomic conditions may have also favoured the preservation of batoid species with very small teeth (around or less than 1mm), whereas the other Eocene deposits appear strongly biased towards taxa with larger teeth due to collecting biases. However, we do not exclude that some of teeth from Boreal and Tethyan concentration deposits might belong to genera erected for Bolca specimens (e.g. *Tethytrygon*, *Titanonarke*, *Promyliobatis*, *Pseudorhinobatos*). Although taphonomic and collecting biases have to be considered, the different taxonomic composition of the Bolca assemblages compared with other Eocene deposits might represent, on the contrary, a true biological signal largely related to the different palaeoenvironmental conditions, supporting the hypothesis of the existence of at least two isolated tropical, inner shelf, shallow‐water communities associated with coral reefs in the western Tethys during the early Eocene.

### Palaeoecological and palaeoenvironmental implications

Sedimentological, palaeontological and geochemical evidences indicate that the Pesciara and Monte Postale fossiliferous deposits originated in two different tropical, coastal, shallow marine palaeobiotopes in the inner portion of the Lessini Shelf where they were in some ways associated to coral reef systems and close to emerged areas (Landini & Sorbini 1996; Papazzoni & Trevisani 2006; Schwark *et al*. 2009; Papazzoni *et al*. 2014a, 2014b, 2017). Because of their high taxonomic diversity and based on auto‐ and synecological considerations, the bony fish fauna is clearly indicative of a heterogeneous shallow marine context, characterized by lagoons, sand bottoms, seagrass beds, coral reefs and influenced by the open sea and emerged areas (Landini & Sorbini 1996; Carnevale *et al*. 2014; Marramà *et al*. 2016c). The bathymetric analysis of the Pesciara and Monte Postale chondrichthyan assemblages suggests depths reaching 40–50 m (Fig. 3), supporting the scenario of an inner shelf environment (Landini & Sorbini 1996; Papazzoni & Trevisani 2006; Papazzoni *et al*. 2017). Support of this bathymetric estimate is derived from the dominance of benthic batoids, and in particular of the zanobatid *Plesiozanobatus* at the Pesciara site, and the platyrhinid *Eoplatyrhina* at Monte Postale, whose extant relatives usually prefer waters shallower than 40‐50 m deep (Fig. 3). Although some of the taxa (*Galeorhinus*, *Brachycarcharias*, *Titanonarke*) have extant relatives also occurring in deep waters, none of them is exclusively associated with deep‐water environments.

Based on sedimentological evidence and ecological requirements of the fossil fish taxa, Landini & Sorbini (1996) recognized three main ecological categories for the fishes from the Pesciara site: (1) the sea/sand‐grass bed assemblage characterized by benthic species closely associated with the sediment (including platyrhinids and dasyatids); (2) the true coral assemblage (no chondrichthyans); (3) the perireefal/pelagic assemblage (including all the sharks). It must be pointed out that Landini & Sorbini (1996) did not take into account several other chondrichthyan taxa known at the time of their publication, and that the chondrichthyan fauna was badly in need of a comprehensive systematic revision. In any case, the composition of the chondrichthyan assemblage is fully consistent with the interpretation of the Pesciara palaeobiotope by these authors. The extant *Galeorhinus* inhabits cool to tropical waters on continental shelves, and juvenile individuals can be relatively common in shallow reef environments (Compagno 2003). Exclusively warm water fossil and living triakids are known as well. Being represented by isolated teeth only, *B. lerichei* and *E. bolcensis* were probably adventitious visitors of the Pesciara palaeobiotope, and likely they were part of the perireefal/pelagic assemblage. As far as the stingray *Tethytrygon* is concerned, modern members of the subfamily Neotrygoninae are demersal, benthic batoids occurring mostly inshore on continental or insular shelves inhabiting warm–temperate and tropical waters often associated with coral reefs (Last *et al*. 2016). Although the peculiar stingray *Lessiniabatis* cannot be linked to any extant family, its body plan is indicative of a pure benthic lifestyle. The same can be assumed for *Plesiozanobatus* (Zanobatidae) and *Eorhinobatos* (Rhinobatidae) whose living relatives inhabit shallow warm–temperate to tropical inshore waters preferably up to 40 and 100 m, respectively (Compagno & Last 1999b; Last *et al*. 2016; Séret 2016), whereas the eagle ray *Promyliobatis* can be regarded as the sole benthopelagic batoid, as living relatives range from the intertidal to the upper slope on soft and hard bottoms, although they mostly occur around coral and rocky reefs, kelp beds, lagoons and bays (Compagno & Last 1999c).

Conversely, all batoids from the Monte Postale association can be considered as benthic species of the sea/sand‐grass bed assemblage, and their presence is consistent with the palaeoenvironmental scenario hypothesized for the Monte Postale palaeobiotope (e.g. Marramà *et al*. 2016c; Papazzoni *et al*. 2017). The extant counterparts of the two *Titanonarke* species, the electric rays of the family Narcinidae, live in tropical inshore to deep waters (up to 1000 m, but usually below 250 m), mostly occurring off soft sandy beaches and in muddy enclosed bays, often associated with coral reefs (Carvalho *et al*. 1999; McEachran & Carvalho 2002). Like *Eoplatyrhina* and *Pseudorhinobatos*, living platyrhinids and rhinobatids are mostly found in warm–temperate to tropical inshore continental waters, occurring in muddy enclosed bays, off sandy beaches, shallow mud bottom and near kelp beds (Compagno & Last 1999a, 1999b), whereas urolophids like *Arechia* have been interpreted to represent temperate to tropical inshore batoids often reaching the upper slope on soft bottoms (Last & Compagno 1999a).

Living chimaeroids mostly inhabit deep waters but some species are known to venture into waters shallower than 40 m to feed or to breed (Bigelow & Schroeder 1953; Last & Stevens 2009). Therefore, the presence of what appears to be a dorsal‐fin spine of *Ischyodus* in the shallow‐water assemblage of the Monte Postale site is not surprising, also considering that Jurassic to Palaeogene chimaeroids are often found in shallow‐water contexts (e.g. Stahl 1999; Kriwet & Gaździcki 2003; Kriwet & Klug 2011).

**Fig. 5:**
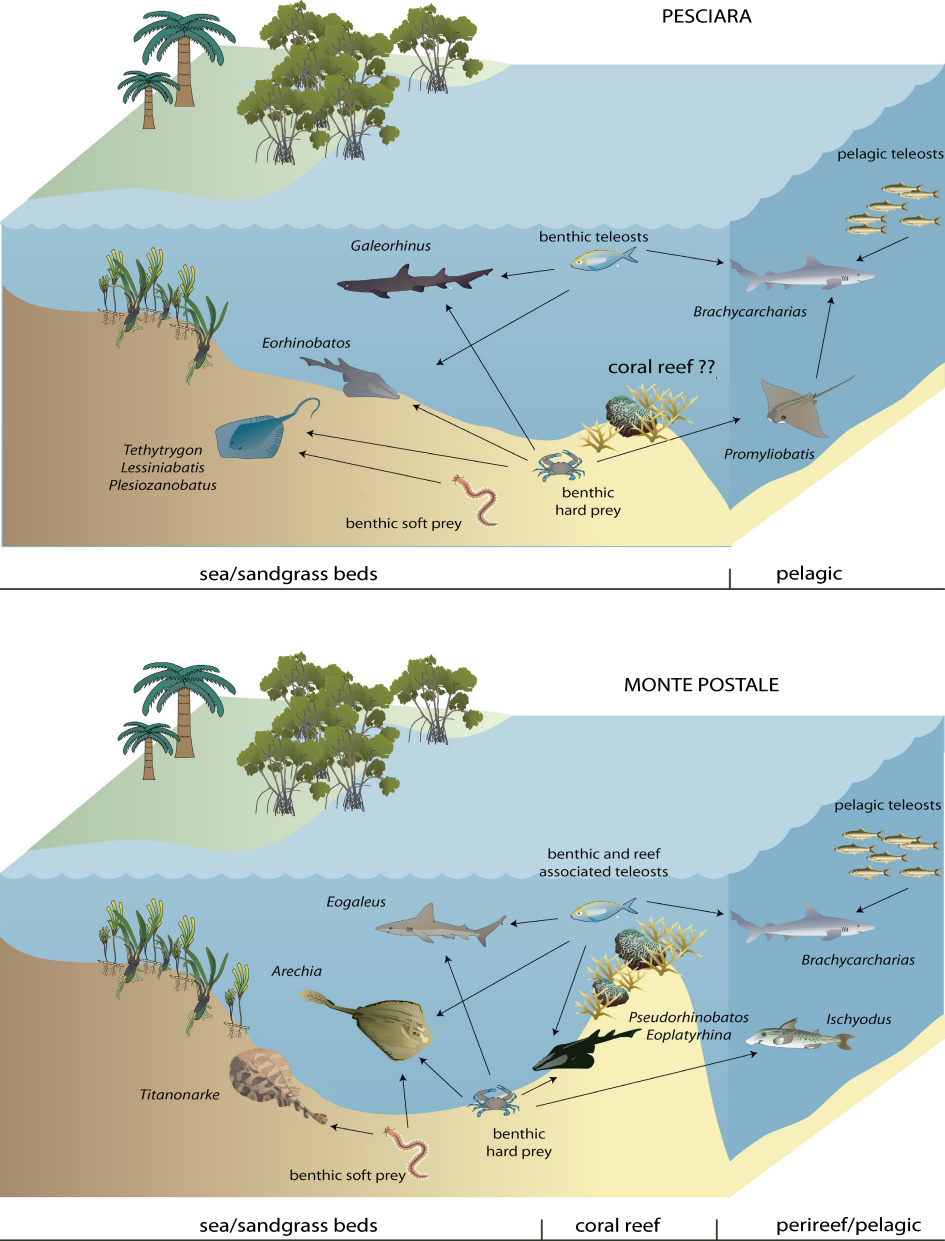
Schematic presentation of the ecological role of chondrichthyan fishes in the palaeobiotopes of Pesciara and Monte Postale sites. Arrows indicate the direct trophic interactions. Images are courtesy of the Integration and Application Network, University of Maryland Center for Environmental Science (ian.umces.edu/symbols/), and Harvard Library, © [2020] President and Fellows of Harvard College, licenced under a Creative Commons Attribution 4.0 International License.

Although it is difficult to define the precise ecological role played by the Pesciara and Monte Postale chondrichthyan faunas in the western Tethys, we can speculate about their trophic significance and interactions thanks to the excellent preservation of their skeletal remains and their overall similarity to extant taxa (Fig. 5). The Bolca assemblages are dominated by durophagous/cancritrophic taxa, particularly benthic batoids (Figs 1, 2, 5; Table 1). Their potential benthic prey includes several crustaceans (including isopods, stomatopods and decapods), and a variety of mollusc species (bivalves, gastropods and cephalopods) (Dominici 2014; Giusberti *et al*. 2014; Pasini *et al*. 2019). The relative abundance of the piscivores, mostly represented by sharks, could be related to the remarkably diverse teleostean assemblage of the Bolca deposits (Carnevale *et al*. 2014). It is important to note that all shark species of Bolca are relatively small (less than 200 cm) and presumably did not represent apex predators but instead functioned as meso‐predators (see Roff *et al*. 2016) along with some of the largest teleosts and rays. Feeders on benthic soft prey are represented at Monte Postale site by the narcinid electric ray *Titanonarke*, and possibly by the urolophid *Arechia*. Their soft prey might have included polychaete species found in both Pesciara and Monte Postale deposits (Alessandrello 1990; Giusberti *et al*. 2014). The stomach content of the electric ray *Titanonarke* also documents the origin of a new feeding mode in chondrichthyans, that is, foraminiferivory (Marramà *et al*. 2018c). The absence of large predatory eurytrophic sharks and filter feeding microphagous taxa may support the assumption of the absence of deep high‐productive environments in the proximity of the original palaeobiotope, the absence of adequate trophic resources or palaeoenvironmental conditions that precluded their access.

The absence of skates (i.e. the Rajiformes *sensu* Naylor *et al*. 2012) in both the Bolca deposits is also consistent with their environmental affinities. Skates are benthic batoids occurring worldwide from continental and insular shelves to abyssal depths, from temperate to cold waters (Last & Compagno 1999b; Last *et al*. 2016). However, they are rare or completely absent in tropical shallow waters, especially around coral reefs (McEachran & Dunn 1998; McEachran & Carvalho 2002) where their ecological role is possibly replaced by stingrays.

Representatives of the rhinopristiform families Pristidae and Rhinidae also are absent in Bolca, although modern sawfishes and wedgefishes typically live in tropical shallow waters (Last *et al*. 2016) and their fossils occur worldwide at least since the early Eocene (e.g. Wueringer *et al*. 2009; Cappetta 2012; Collareta *et al*. 2020). The absence of pristids and rhinids in the Bolca deposits might be related to the coral reef setting hypothesized for Bolca, which might have favoured rhinobatids over other rhinopristiform families, which due to their peculiar foraging strategies (e.g. Wueringer *et al*. 2009) might prove less likely to be associated with reef environments.

## Conclusions

In this paper, the diversity, palaeoecological role and the palaeoenvironmental significance of the chondrichthyan assemblages of the two main sites of the Bolca Lagerstätte have been definitively assessed. Both the assemblage of the Pesciara and Monte Postale sites are characterized by a predominance of benthic batoids with durophagous/cancritrophic feeding preferences, followed by piscivores, especially selachians. Soft‐prey feeders (e.g. torpediniforms) appear to be exclusive of the Monte Postale assemblage. The habitat preferences and palaeobathymetric analyses support the hypothesis that the early Eocene fish communities of the Bolca Lagerstätten inhabited the western Tethyan tropical shallow marine waters of the inner‐middle portion of the Lessini Shelf, which were surrounded by coral reefs.

## Data availability statement

The data that support the findings of this study are openly available in Figshare at http://doi.org/10.6084/m9.figshare.14095695.
